# Psychological and physical effects of short-term discontinuation of feminizing gender-affirming hormone therapy among older transgender women: a within-subject clinical trial

**DOI:** 10.1093/humrep/deag087

**Published:** 2026-05-30

**Authors:** J O van Heesewijk, R E S Geels, M den Heijer, K M A Dreijerink, B P C Kreukels

**Affiliations:** Center of Expertise on Gender Dysphoria, Amsterdam UMC, Location VUmc, Amsterdam, The Netherlands; Department of Endocrinology and Metabolism, Amsterdam UMC, Location VUmc, Amsterdam, The Netherlands; Amsterdam Public Health (APH) Research Institute, Amsterdam UMC, Location VUmc, Amsterdam, The Netherlands; Department of Medical Psychology, Amsterdam UMC, Location VUmc, Amsterdam, The Netherlands; Center of Expertise on Gender Dysphoria, Amsterdam UMC, Location VUmc, Amsterdam, The Netherlands; Department of Endocrinology and Metabolism, Amsterdam UMC, Location VUmc, Amsterdam, The Netherlands; Amsterdam Gastroenterology Endocrinology Metabolism Research Institute, Amsterdam UMC, Location VUmc, Amsterdam, The Netherlands; Center of Expertise on Gender Dysphoria, Amsterdam UMC, Location VUmc, Amsterdam, The Netherlands; Department of Endocrinology and Metabolism, Amsterdam UMC, Location VUmc, Amsterdam, The Netherlands; Amsterdam Gastroenterology Endocrinology Metabolism Research Institute, Amsterdam UMC, Location VUmc, Amsterdam, The Netherlands; Center of Expertise on Gender Dysphoria, Amsterdam UMC, Location VUmc, Amsterdam, The Netherlands; Department of Endocrinology and Metabolism, Amsterdam UMC, Location VUmc, Amsterdam, The Netherlands; Amsterdam Gastroenterology Endocrinology Metabolism Research Institute, Amsterdam UMC, Location VUmc, Amsterdam, The Netherlands; Center of Expertise on Gender Dysphoria, Amsterdam UMC, Location VUmc, Amsterdam, The Netherlands; Amsterdam Public Health (APH) Research Institute, Amsterdam UMC, Location VUmc, Amsterdam, The Netherlands; Department of Medical Psychology, Amsterdam UMC, Location VUmc, Amsterdam, The Netherlands

**Keywords:** transgender aging, gender-affirming hormone therapy, estradiol deprivation, sleep quality, psychological well-being

## Abstract

**STUDY QUESTION:**

What are the psychological and physical effects of short-term discontinuation and subsequent reinitiation of feminizing gender-affirming hormone therapy (fGAHT) in older transgender women receiving long-term therapy?

**SUMMARY ANSWER:**

Short-term fGAHT discontinuation increased estradiol deprivation-related symptoms while reinitiation showed opposite effects, and psychological well-being was not affected.

**WHAT IS KNOWN ALREADY:**

With growing numbers of older transgender individuals, questions arise regarding the necessity of long-term continuation of fGAHT, particularly since cisgender women have low estradiol concentrations after menopause. However, data on the effects of fGAHT discontinuation in older transgender women are lacking.

**STUDY DESIGN, SIZE, DURATION:**

This within-subject clinical trial included 19 participants and was conducted in 2024 and 2025.

**PARTICIPANTS/MATERIALS, SETTING, METHODS:**

Transgender women aged ≥55 years (median age 66 years), on fGAHT for ≥5 years (median 23 years), were recruited from the Center of Expertise on Gender Dysphoria, Amsterdam, the Netherlands. Estradiol deprivation-related symptoms, sleep quality, quality of life, mental health, and body image were assessed via questionnaires at baseline, 12 weeks after fGAHT discontinuation, and 12 weeks after reinitiation. Outcomes were analyzed using multilevel models. Differences were considered substantial based on 95% CIs and ≥10% change between measurements. Perceived psychological and physical changes and willingness to permanently discontinue fGAHT were also assessed via open-ended questions.

**MAIN RESULTS AND THE ROLE OF CHANCE:**

Experiences varied, but after discontinuation, unfavorable mean changes were found for somato-vegetative symptoms (β 0.4, 95% CI −0.7 to 1.5, %Δ 11.5), sleep disturbance (OR 1.6, 95% CI 0.2 to 10.3, %Δ 10.7), and happiness to a lesser extent (β −0.5, 95% CI −0.9 to −0.1, %Δ −6.4). Reinitiation showed favorable changes for somato-vegetative and total estradiol deprivation-related symptoms (β −0.8, 95% CI −1.9 to 0.3, %Δ −22.1; β −3.0, 95% CI −4.8 to −1.2, %Δ −36.3), total sleep quality (β −0.7, 95% CI −1.5 to 0.2, %Δ −10.3), sleep onset latency (β −2.8, 95% CI −8.1 to 2.5, %Δ −14.9), and sleep disturbance (OR 0.1, 95% CI 0.0 to 1.0, %Δ −46.6). No mean differences were found for depressive symptoms, anxiety, quality of life, or body satisfaction. Perceived psychological and physical changes were diverse and generally aligned with known menopause-related changes. One participant wished to permanently stop fGAHT.

**LIMITATIONS, REASONS FOR CAUTION:**

The study was not blinded or placebo-controlled, which may have introduced expectancy bias. The small clinical sample may limit generalizability to the broader population of transgender women.

**WIDER IMPLICATIONS OF THE FINDINGS:**

Transgender women, like cisgender women, experience symptoms in response to estradiol deprivation, which may support the continuation of fGAHT in older age. This study did not identify psychological or body image barriers to discontinuation for those who wish to stop.

**STUDY FUNDING/COMPETING INTEREST(S):**

Funded by an Amsterdam UMC PhD scholarship to J.O.v.H. and by departmental funds from the Department of Endocrinology and Metabolism, Amsterdam UMC, location VUmc. K.M.A.D. declares receipt of consulting fees from Recordati, paid to their institution. The other authors declare no competing interests.

**TRIAL REGISTRATION NUMBER:**

This study was registered in the EU Clinical Trials Register (EUCTR2023-505143-39-00).

## Introduction

With growing academic interest in minority groups and aging populations, research on the health and well-being of older transgender adults is slowly increasing. However, significant gaps remain in our understanding of this group’s health needs and outcomes, highlighting the urgency for further study in this growing population (Fredriksen-Goldsen *et al.*, 2014; den Heijer *et al.*, 2017; Fabbre and Siverskog, 2019, Flatt *et al.*, 2022; van Heesewijk *et al.*, 2023; Toze *et al.*, 2024). A recent call to action urged clinicians, academics, and policy makers to address a range of knowledge gaps and invest in the well-being of older transgender adults (Green *et al.*, 2025).

A particularly important topic is the long-term use of gender-affirming hormone therapy (GAHT). In cisgender women, menopause around ages 45–55 leads to a sharp decline in estradiol concentrations, prompting questions about the need for continuation of feminizing GAHT (fGAHT) in transgender women (male sex-assigned at birth, feminine gender identity) of similar age (Davis and Baber, 2022). fGAHT typically involves estradiol therapy, with anti-androgens used before gonadectomy and if desired. From age 45 onward, it is recommended that the route of estradiol therapy be changed to transdermal application (Hembree *et al.*, 2017; Houlberg, 2019; Coleman *et al.*, 2022). However, there are no evidence-based guidelines for fGAHT continuation in older individuals, due to limited data on its long-term risks and benefits. In practice, fGAHT is often continued into older age, and the dose can be reduced from age 50 onward, although some patients may wish to permanently discontinue fGAHT. In the Netherlands, such choices are typically guided by shared decision-making. When contraindications such as hormone-sensitive breast cancer or other risk factors are present, clinicians may advise to reduce the dose or discontinue fGAHT altogether.

Understanding the benefits of fGAHT for transgender women’s well-being is essential for estimating the potential impact of discontinuation. The primary aim of (f)GAHT is to align physical appearance with gender identity, thereby reducing gender incongruence, a medically necessary intervention in cases of significant distress (American Psychiatric Association, 2022; Coleman *et al.*, 2022). Beyond this, fGAHT has been shown to improve self-esteem, body image, psychological distress, mental health, and, although less consistently, quality of life (Costa and Colizzi, 2016; Doyle *et al.*, 2023; Meneguzzo *et al.*, 2024). Given that older transgender adults, particularly transgender women, experience poorer mental and social health compared to cisgender peers (Fredriksen-Goldsen *et al.*, 2014; van Heesewijk *et al.*, 2025), these outcomes are especially relevant when considering fGAHT discontinuation.

Literature addressing menopausal and estradiol deprivation experiences in transgender adults is scarce, with transgender women particularly underrepresented. A qualitative survey study included one older transgender woman who reported experiencing estradiol deprivation-related symptoms after (unwanted) fGAHT interruptions, and noted that transgender women are often not believed to experience such symptoms and are left out of discussions and considerations about menopause (Toze and Westwood, 2025). The latest version of the NICE menopause guideline includes transgender men and non-binary people assigned female at birth not on GAHT, but omits transgender women (NICE, 2024). A mixed-methods study found that transgender women’s perspectives on menopause and long-term fGAHT continuation vary; some view menopause as irrelevant for themselves, some expect dose reductions, and others are uncertain about what to expect (Mohamed and Hunter, 2019). Concerns were raised about long-term fGAHT effects, being forced to stop, and adverse effects of discontinuation.

Potential effects of fGAHT discontinuation can be inferred from menopausal experiences in cisgender women, who often report a variety of symptoms due to estradiol deprivation such as vasomotor symptoms (e.g. night sweats, hot flushes), sleep problems (e.g. longer time to fall asleep, sleep disturbances), and poorer mental health (e.g. more depressive and anxiety symptoms) (Sun *et al.*, 2014; Davis *et al.*, 2015; Davis and Baber, 2022; Hogervorst *et al.*, 2022; Zeng *et al.*, 2025). Menopause hormone therapy, consisting of estradiol supplementation (combined with progesterone or progestins for women with a uterus), effectively alleviates vasomotor symptoms and may also improve sleep quality and mental health (Davis *et al.*, 2015; Baber *et al.*, 2016; Hogervorst *et al.*, 2022). However, as it remains unknown to what extent these health aspects are affected by fGAHT discontinuation in transgender women, we aimed to examine psychological and physical effects of short-term discontinuation and subsequent reinitiation of fGAHT among older transgender women receiving long-term therapy.

## Materials and methods

### Study design and methodological considerations

This within-subject clinical trial was conducted in 2024 and 2025 at the Center of Expertise on Gender Dysphoria (CEGD) at Amsterdam UMC, location VUmc. The trial design and outcome measures were initially proposed by the authors, addressing gaps in the literature and questions from clinicians and patients about continuing GAHT in older age. Two older transgender men from the authors’ networks and three older transgender women from the CEGD patient advisory board were consulted in individual, unrecorded online meetings about the feasibility and relevance of the study and its potential outcomes. The women considered the study highly relevant, especially in relation to menopause in cisgender women, and recommended extending the discontinuation period from the initially proposed 8–12 weeks to better assess physical changes, but felt stopping for longer than 3 months was not feasible. They also suggested adding open-ended questions about psychological and physical changes, as well as items on energy, sex drive, and skin and hair. The men did not find the study relevant for themselves, as stopping testosterone would not mimic the physiology of older cisgender men. They believed most men would be unwilling to discontinue testosterone, but suggested considering dose reduction instead. They did express interest in changes in sleep apnea.

Given these insights, we chose to focus on 12 weeks of fGAHT discontinuation in transgender women only. While dose reduction in men could also be relevant, we expected that, compared to complete discontinuation, this would result in effects that are too small to observe within a short timeframe. Balancing feasibility with the ability to detect changes, we included a fGAHT reinitiation follow-up period, allowing us to assess the effects of fGAHT changes in two ways to strengthen our findings without extending the discontinuation period. Therefore, the study included three outpatient clinic visits over approximately 6 months per participant: a baseline visit (*t* = 0), a follow-up 12 weeks (range 10–14) after fGAHT discontinuation (*t* = 1), and 12 weeks (range 10–14) after reinitiation of the original fGAHT dosage (see Supplementary Fig. S1). Each visit lasted about 1.5 h and comprised weight and blood pressure measurements, a consultation with the study physician, laboratory assessments, and completion of questionnaires, including open-ended questions, entered into the Castor EDC clinical research data platform. Clinical data on endocrine and cardiometabolic changes will be reported separately.

### Ethical approval

This study was conducted in accordance with the Declaration of Helsinki and was approved by the Medical Ethics Review Board of Amsterdam UMC, location VUmc (EU CT 2023-505143-39-00). Written informed consent was obtained from all participants.

### Participants

Patients were recruited from the CEGD outpatient clinic if they were aged 55 years or older, had undergone gonadectomy, were currently receiving fGAHT for at least 10 years, and were in active treatment at the CEGD (i.e. clinical appointment within the preceding 3 years). Insufficient proficiency in Dutch was an exclusion criterion, given that all questionnaires and study materials were in Dutch. As recruitment progressed more slowly than anticipated, eligibility criteria were broadened to include patients who had received fGAHT for at least 5 years. Eligible participants were contacted by telephone after their treating physician had obtained their permission to be contacted. Additional recruitment included participants from a previous study who had consented to future contact (van Heesewijk *et al.*, 2023). After a minimum 2-week consideration period, patients were contacted again by telephone to schedule the first study visit with one of the study physicians (J.O.v.H., K.M.A.D.). All participants received transdermal estradiol monotherapy, including estradiol patches (50–100 μg/24 h, twice weekly; Systen^®^/Sandoz^®^), estradiol gel (1.25–5.0 mg/day, daily; Oestrogel^®^), and estradiol spray (1.53–4.59 mg/day, daily; Lenzetto^®^). For an overview of regimens and dosages, see Table 1.

**Table 1.** Baseline characteristics.

Baseline characteristics	N = 19
Age in years (median (IQR))	66 (63–68)
Age range	59–78
fGAHT duration in years (median (IQR))	23 (17–33)
fGAHT duration range	8–48
Vaginoplasty* (no. (%))	17 (89)
Orchiectomy (no. (%))	2 (11)
Non-white ethnicity (no. (%))	1 (5)
Route of estradiol administration (no. (%))	
Estradiol patch	7 (37)
50 μg/24 h	4 (21)
100 μg/24 h	3 (16)
Estradiol gel	8 (42)
1.25 mg/day	4 (21)
2.50 mg/day	3 (16)
5.00 mg/day	1 (5)
Estradiol spray	4 (21)
1.53 mg/day	1 (5)
3.06 mg/day	2 (11)
4.59 mg/day	1 (5)

* Surgical procedure that includes removal of the testes.

IQR: interquartile range; fGAHT: feminizing gender-affirming hormone therapy.

### Outcome variables

Questionnaires and subscores were selected based on their anticipated sensitivity to changes following short-term fGAHT discontinuation, their prior administration in older populations (except for the Body Image Scale (BIS)), and psychometric properties, including good internal consistency and test-retest reliability (Lindgren and Pauly, 1975; Buysse *et al.*, 1989; Heinemann *et al.*, 2004; Skevington *et al.*, 2004; Cella *et al.*, 2010; Tov *et al.*, 2021). However, these questionnaires have not been specifically validated for older transgender populations.

#### Menopause Rating Scale

The Menopause Rating Scale (MRS) measures menopause-specific health-related quality of life (QoL) through questions about current menopausal symptoms (Hauser *et al.*, 1994; Heinemann *et al.*, 2004), hereafter referred to as *estradiol deprivation-related symptoms*. It comprises 11 items scored from 0 (no symptoms) to 4 (very severe symptoms), which are summed to provide psychological (range 0–16), somato-vegetative (including vasomotor symptoms, sleep problems, and muscle and joint soreness; 0–16), and urogenital (0–12) subscales, as well as a total score (0–44), with higher scores indicating more symptoms. For this study, the total and somato-vegetative scores were used. The item on vaginal dryness was excluded, as it was not applicable to all participants. The total score range was 0–40 for this study.

#### Pittsburgh Sleep Quality Index

The Pittsburgh Sleep Quality Index (PSQI) assesses *sleep quality* through 19 items on sleep habits and problems in the past month (Buysse *et al.*, 1989). These items generate seven component scores, including subjective sleep quality, sleep medication use, daytime dysfunction, and sleep onset latency, duration, efficiency and disturbance. Components are rated from 0 (no difficulty) to 3 (severe difficulty), which are summed to generate a total score (0–21). Higher scores reflect poorer sleep quality. In this study, total scores were used, along with continuous (rather than ordinal 0–3) measures for the components sleep onset latency, recorded as the average time to fall asleep in minutes, and sleep efficiency, calculated as the percentage of time spent asleep relative to the total time dedicated to sleep, as described in Morssinkhof *et al.* (2023). Additionally, the component sleep disturbance was dichotomized, grouping no and mild difficulty, and moderate and severe difficulty, as the ordinal models did not converge.

#### Patient-Reported Outcomes Measurement Information System depression and anxiety

*Depressive and anxiety symptoms* over the past 7 days were assessed using the Patient-Reported Outcomes Measurement Information System (PROMIS) Short Form v1.0–8b for depression and 8a for anxiety (Cella *et al.*, 2010). Each questionnaire includes eight items, scored from 1 (never) to 5 (always). These are summed to provide a total score ranging from 8 to 40, with higher scores indicating more symptoms.

#### Cantril ladder

The modified Cantril ladder is a single-item visual scale, which was used to measure current feelings of *happiness*, ranging from 0 (bad) to 10 (very good) (Cantril, 1965).

#### World Health Organization QoL-BREF

The World Health Organization (WHO) QoL-BREF is a short-form version of the WHOQoL-100, assessing *QoL* over the past 2 weeks across four domains: physical health, psychological health, social relationships, and environment (The Whoqol G, 1998). The domains in the short form consist of 24 items rated on a five-point scale, with higher scores reflecting better QoL. In this study, the physical health (range 7–35) and psychological health (range 6–30) domain scores were calculated by summing the relevant items.

#### Body Image Scale

*Body image* was measured using the Body Image Scale (BIS), which asks participants to rate their satisfaction with 34 physical features or body parts. The scale, adapted from Lindgren and Pauly (1975), ranges from 1 (very satisfied) to 5 (very unsatisfied), with a score of 6 indicating ‘not applicable’ for sex-specific characteristics. Only relevant items were included for each participant, and participants could skip any items they preferred not to answer due to the sensitive content of the questionnaire. Lindgren and Pauly (1975) calculated a mean total score and three subscores for primary, secondary, and neutral characteristics. In the present study, we calculated total scores and subscores for secondary sex characteristics, provided at least 50% of items were completed. Higher average BIS scores reflect higher body dissatisfaction.

##### Open-ended questions

A question about perceived skin changes was added to Castor EDC at both follow-up visits, asking participants whether they had noticed any skin changes after discontinuation or reinitiation of fGAHT. If so, participants could describe the changes in an open-ended field. Additionally, study physicians inquired about any psychological or physical changes perceived after discontinuation and reinitiation, participants’ satisfaction with these changes, and whether they would consider permanently stopping fGAHT based on their experiences in the study. Of note, permanent discontinuation was not actively offered as an option, but was possible at the participant’s request. The physicians’ notes from these consultations were subsequently entered into Castor.

### Statistical analyses

Descriptive statistics were calculated for each time point, with medians and interquartile ranges (IQRs) reported for continuous outcomes and percentages for categorical outcomes. Open-ended responses and physicians’ notes regarding perceived psychological and physical changes were categorized, with skin changes reported separately from other physical changes, as these were specifically prompted, while changes noted by physicians were spontaneously reported. Percentages were calculated for each category, as well as for physicians’ notes regarding considerations about discontinuing fGAHT. Continuous outcomes were analyzed using linear mixed models, and the dichotomous outcome was analyzed with logistic mixed models, with observations clustered within participants. Analyses were performed in Stata (version 18.5), with *t* = 1 set as the reference category comparing *t* = 0 to *t* = 1 for discontinuation effects, and *t* = 1 to *t* = 2 for reinitiation effects. Differences were considered substantial based on their size and the 95% CI. As the minimal important change has not been established for these outcome measures in this population, we pragmatically defined substantial differences as those exceeding 10%, irrespective of statistical significance. Given the small sample size and potential impact of outliers, sensitivity analyses were conducted excluding outliers (>1.5×IQR). Missing data occurred only for the BIS (16%), as these items were optional and sometimes not applicable. Data were not imputed, as mixed models appropriately handle missing values (Twisk *et al.*, 2013).

## Results

### Participant characteristics

From 2024, 145 transgender women were invited to participate in this study at the CEGD. Thirty-five patients were not eligible, mostly because they were not in treatment at the CEGD anymore. Thirty patients did not want to stop estradiol therapy, 25 could not be reached, 24 considered the practical burden of participating too high, and 11 did not participate for unknown or other reasons. In total, there were 20 participants (participation rate 20/85 = 0.24), of which one discontinued the study after 1 month due to an adverse event (i.e. gastric pain) probably unrelated to fGAHT discontinuation. She was excluded from the analyses as only one measurement was completed. Baseline characteristics of all 19 participants are shown in Table 1. The median age was 66 years (range 59–78 years), and the median fGAHT duration was 23 years (8–48 years). All participants underwent gonadectomy as per the inclusion criteria; the majority underwent vaginoplasty (N = 17; 89%), which includes removal of the testes, and two underwent orchiectomy only (11%). One participant (5%) was of non-white ethnicity. No relevant changes in medication use (e.g. antidepressants) were observed between time points.

### Psychological and physical effects

**Figure 1.**
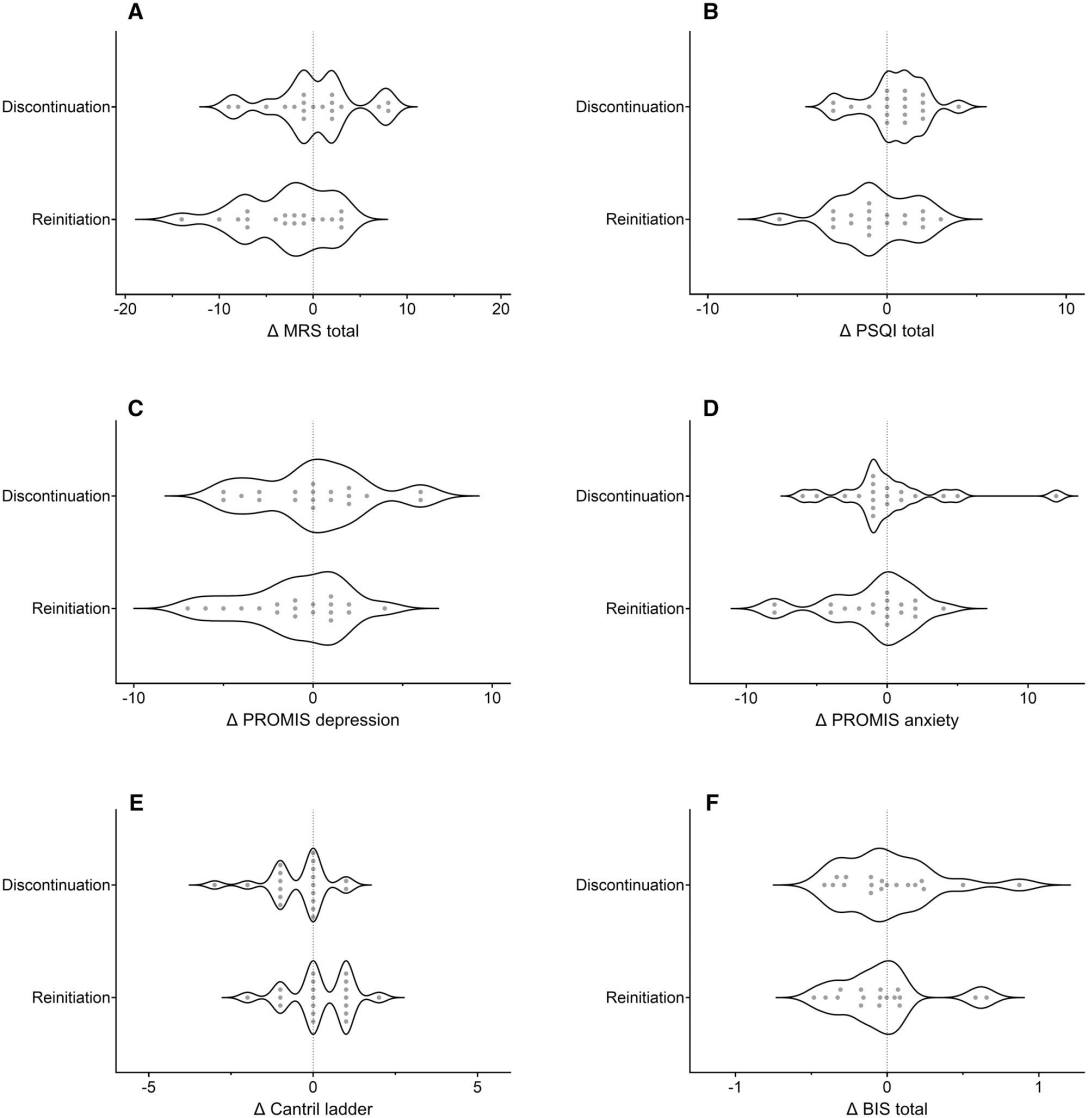
**(A-F) Violin plots showing the variation in change over time on total scores.** Each point represents the change score of one participant, with ‘discontinuation’ showing the difference between *t* = 0 and *t* = 1, and ‘reinitiation’ between *t* = 1 and *t* = 2. The dotted lines at zero indicate no difference. Δ: score differences; MRS: Menopause Rating Scale; PSQI: Pittsburgh Sleep Quality Index; PROMIS: Patient-Reported Outcomes Measurement Information System; BIS: Body Image Scale.

Table 2 shows the estimated means per time point of all main study outcomes, and estimated mean differences including percentage change between *t* = 0 and *t* = 1, and *t* = 1 and *t* = 2. Sensitivity analyses are shown in Supplementary Table S1. Figure 1 and Supplementary Fig. S2 display the variation in change over time on total scores and subscores, respectively, to illustrate within-group variability beyond the estimated mean differences presented in Table 2. The estimated mean of total estradiol deprivation-related symptoms did not substantially increase following discontinuation, but experienced changes varied (Fig. 1A). A substantial increase in somato-vegetative symptoms after fGAHT discontinuation was found (β 0.4, 95% CI −0.7 to 1.5, %Δ 11.5; Supplementary Fig. S2A), as well as substantially decreased somato-vegetative and total estradiol deprivation-related symptoms after fGAHT reinitiation (β −0.8, 95% CI −1.9 to 0.3, %Δ −22.1; β −3.0, 95% CI −4.8 to −1.2, %Δ −36.3).

**Table 2.** Estimated means, mean differences, and percentage change between measurements following short-term discontinuation and subsequent reinitiation of feminizing gender-affirming hormone therapy.

	Baseline, *t* = 0 (N = 19)	Following fGAHT discontinuation, *t* = 1 (N = 19)			Following fGAHT reinitiation, *t* = 2 (N = 19)		
Outcome variables	Estimated mean (95% CI)	Estimated mean (95% CI)	Estimated mean difference *t* = 0 to *t* = 1 (95% CI)	% change	Estimated mean (95% CI)	Estimated mean difference *t* = 1 to *t* = 2 (95% CI)	% change
Estradiol deprivation-related symptoms—Total	8.1 (5.8 to 10.3)	8.3 (6.0 to 10.5)	0.2 (−1.6 to 2.0)	2.6	5.3 (3.0 to 7.5)	−**3.0 (**−**4.8 to** −**1.2)**	−**36.3**
Somato-vegetative symptoms	3.2 (2.1 to 4.3)	3.6 (2.5 to 4.7)	0.4 (−0.7 to 1.5)	**11.5**	2.8 (1.7 to 3.9)	−0.8 (−1.9 to 0.3)	−**22.1**
Sleep quality—Total	6.2 (4.5 to 7.9)	6.6 (5.0 to 8.3)	0.4 (−0.4 to 1.3)	6.8	5.9 (4.3 to 7.6)	−0.7 (−1.5 to 0.2)	−**10.3**
Sleep onset latency	22.1 (14.9 to 29.4)	18.4 (11.2 to 25.7)	−3.7 (−9.0 to 1.6)	−**16.6**	15.7 (8.4 to 22.9)	−2.8 (−8.1 to 2.5)	−**14.9**
Sleep efficiency	77.9 (70.0 to 85.8)	74.2 (66.3 to 82.1)	−3.7 (−11.0 to 3.5)	−4.8	76.9 (69.0 to 84.8)	2.7 (−4.6 to 9.9)	3.6
Sleep disturbance	0.5 (0.3 to 0.8)	0.6 (0.4 to 0.8)	1.6* (0.2 to 10.3)	**10.7**	0.3 (0.1 to 0.5)	**0.1** * **(0.0 to 1.0)**	−**46.6**
Depressive symptoms	13.6 (11.3 to 15.9)	13.6 (11.4 to 15.9)	0.1 (−1.3 to 1.4)	0.4	12.6 (10.3 to 14.9)	−1.1 (−2.4 to 0.3)	−7.7
Anxiety symptoms	13.6 (11.5 to 15.8)	13.8 (11.6 to 15.9)	0.2 (−1.6 to 2.0)	1.2	12.8 (10.6 to 14.9)	−1.0 (−2.8 to 0.8)	−7.3
Happiness	7.4 (6.8 to 7.9)	6.9 (6.4 to 7.4)	−**0.5 (**−**0.9 to** −**0.1)**	−6.4	7.1 (6.6 to 7.6)	0.2 (−0.2 to 0.6)	3.1
QoL—Physical health	25.8 (23.4 to 28.3)	25.4 (23.0 to 27.8)	−0.5 (−1.7 to 0.7)	−1.8	25.8 (23.4 to 28.2)	0.4 (−0.8 to 1.6)	1.7
QoL—Psychological health	21.2 (19.7 to 22.6)	21.2 (19.7 to 22.6)	0.0 (−0.8 to 0.8)	0.0	21.2 (19.8 to 22.6)	0.1 (−0.8 to 0.9)	0.2
Body image—Total	2.6 (2.4 to 2.8)	2.6 (2.4 to 2.9)	0.0 (−0.1 to 0.2)	0.7	2.6 (2.4 to 2.8)	−0.1 (−0.2 to 0.1)	−2.0
Body image—Secondary sex characteristics	2.7 (2.5 to 2.9)	2.7 (2.5 to 3.0)	0.0 (−0.1 to 0.2)	0.3	2.7 (2.4 to 2.9)	0.0 (−0.2 to 0.1)	−1.5

Bold = significance based on 95% CI or >10% change in estimated means between measurements.

Outcome variables were measured using the following questionnaires, listed in order from top to bottom: Menopause Rating Scale, Pittsburgh Sleep Quality Index, PROMIS depression Short Form v1.0 8b, PROMIS anxiety Short Form v1.0 8a, Cantril ladder, World Health Organization QoL-BREF, Body Image Scale.

* Dichotomous outcome, therefore, odds ratios are shown.

fGAHT: feminizing gender-affirming hormone therapy; QoL: quality of life.

Discontinuation did not result in substantially poorer total sleep quality in the main analyses (Fig. 1B), but did after removing outliers (β 0.6, 95% CI −0.2 to 1.5, %Δ 11.0), and improved after fGAHT reinitiation (β −0.7, 95% CI −1.5 to 0.2, %Δ −10.3). Sleep onset latency substantially decreased after discontinuation (β −3.7, 95% CI −9.0 to 1.6, %Δ −16.6; Supplementary Fig. S2C) and reinitiation (β −2.8, 95% CI −8.1 to 2.5, %Δ −14.9). The odds of experiencing sleep disturbance were 1.6 times higher after fGAHT discontinuation (95% CI 0.2 to 10.3, %Δ 10.7) and decreased by 90% after reinitiation (95% CI 0.0 to 1.0, %Δ −46.6). No substantial differences in sleep efficiency were observed at any time point, and variation is shown in Supplementary Fig. S2D.

On average, depressive and anxiety symptoms did not change substantially at either follow-up period (Fig. 1C and D). A minor decrease in happiness was found after discontinuation (β −0.5, 95% CI −0.9 to −0.1, %Δ −6.4; Fig. 1E), which did not significantly or substantially increase following reinitiation. QoL regarding physical and psychological health did not change by the interventions (for variation, see Supplementary Fig. S2E and F). Neither did total body image (Fig. 1F) or body image of secondary sex characteristics (Supplementary Fig. S2B). Sensitivity analyses removing outliers did not change outcomes for any other variables.

### Perceived changes and discontinuation considerations

Table 3 summarizes all perceived psychological and physical changes after discontinuation and reinitiation, as reported either during clinical consultation or in open-ended responses. Most participants experienced at least one psychological (63–68%) and/or physical (84%; including skin and other changes) effect at both follow-up periods. Discontinuation effects varied widely and most frequently included mood swings, rougher, drier or thinner skin, and (nighttime) hot flushes. How these effects were appraised varied as well, with some participants experiencing changes as negative and others reporting only subtle or temporary changes. The majority of discontinuation effects reversed or resolved after fGAHT reinitiation. Some effects persisted, such as weight gain, while others were newly reported after reinitiation, including skin rash, drier skin, and feeling more (positive) emotions. Last, participants varied in considering permanent discontinuation of fGAHT. One participant (5%) chose to stop fGAHT at the end of the study. Nine participants (47%) would only consider permanent discontinuation if medically advised or necessary, as they had concerns about the effects on their mood and/or physical appearance, or noted that they felt better with estradiol than without. Five participants (26%) would not consider stopping fGAHT due to concerns about osteoporosis risk, effects on mood, unknown long-term effects, or because they saw no need to discontinue. Responses were missing for four participants (21%).

**Table 3.** Perceived psychological and physical effects after short-term discontinuation and subsequent reinitiation of feminizing gender-affirming hormone therapy.

fGAHT discontinuation effects (no. (%))	N = 19	fGAHT reinitiation effects (no. (%))	N = 19
Psychological	12 (63)	Psychological	13 (68)
Mood swings, incl. feeling down/crying	5 (26)	Feeling more (positive) emotions	1 (5)
Irritability	4 (21)		
Mentally/cognitively less alert	1 (5)		
Restlessness	1 (5)		
Higher sex drive	1 (5)		
Lower sex drive	1 (5)		
Feeling like ‘part of me is missing’	1 (5)		
Physical	16 (84)	Physical	16 (84)
*Skin*		*Skin*	
Rougher/drier/thinner skin	4 (21)	Skin rash	3 (16)
More (facial) hair growth	3 (16)	Drier skin	1 (5)
Acne	1 (5)		
Vaginal rash/yeast infections stopped	1 (5)		
*Other*		*Other*	
(Nighttime) hot flushes	7 (37)	Weight gain	3 (16)
Tiredness, sleepiness, low energy	3 (16)		
Sleep problems	3 (16)		
(Nighttime) sweatiness	2 (11)		
Nighttime palpitations	1 (5)		
Headache	1 (5)		
Weight loss	1 (5)		
Weight gain	1 (5)		
Less sensitive breasts	1 (5)		
More masculine/older appearance	1 (5)		

Most effects observed after fGAHT discontinuation reversed or resolved following reinitiation. Only outcomes that persisted or were newly reported after reinitiation were included as reinitiation effects. Underlined numbers indicate participants who reported at least one change, *including* those whose changes resolved after reinitiation. fGAHT: feminizing gender-affirming hormone therapy.

## Discussion

This study examined psychological and physical effects of short-term discontinuation and subsequent reinitiation of fGAHT in older transgender women receiving long-term therapy. Discontinuation of fGAHT led to increased somato-vegetative symptoms and sleep disturbances, a shorter time to fall asleep, and slightly lower happiness scores. After removal of outliers, poorer overall sleep quality was also observed. Reinitiation resulted in a decrease in somato-vegetative and overall estradiol deprivation-related symptoms, improved overall sleep quality, fewer sleep disturbances, and a shorter time to fall asleep. No mean changes were observed in depressive and anxiety symptoms, body image, and QoL. Regardless of average differences, the size and direction of changes varied across individuals for all psychological and physical outcomes, highlighting variation in individual experiences.

The diversity in experiences was also reflected in participants’ considerations regarding permanent discontinuation, as well as the perceived psychological and physical changes and their appraisal. One out of 19 participants chose to discontinue fGAHT at the end of the study. The remaining participants, despite varying reasons, were generally unwilling to stop, even those who did not experience negative changes. This suggests a strong attachment to fGAHT that extends beyond psychological or physical side-effects of discontinuation, as well as concerns about the long-term consequences of discontinuation and a perceived lack of relevance. This is consistent with previous findings from a mixed-methods study that examined transgender women’s views on menopause and long-term fGAHT, revealing similar attitudes and uncertainties (Mohamed and Hunter, 2019). The general reluctance to discontinue fGAHT was further reflected by the low participation rate (24%) among 85 eligible patients, with 30 individuals explicitly declining participation because of unwillingness to stop fGAHT. The psychological and physical changes perceived by our participants, such as mood swings and hot flushes, are consistent with those reported in menopausal cisgender women (Davis and Baber, 2022). Similarly, the limited literature on estradiol deprivation in transgender adults describes comparable symptoms, with one older transgender woman reporting vasomotor symptoms as well as sleep and concentration problems following (unwanted) fGAHT interruptions (Toze and Westwood, 2025). Also the perceived skin changes are consistent with findings in postmenopausal women, where estrogen deprivation accelerates skin aging through several mechanisms, including reduced antioxidant protection, resulting in decreased collagen and elasticity, and increased dryness and wrinkling (Thornton, 2013).

Generally, opposite effects were observed after discontinuation and reinitiation, supporting the role of fGAHT in these changes. However, sleep onset latency decreased at both follow-up measurements, which was unexpected. Previous literature indicated that postmenopausal cisgender women experience longer sleep onset latency compared to premenopausal cisgender women (Sun *et al.*, 2014; Zeng *et al.*, 2025), and a younger sample of transgender women showed decreased latency 12 months after starting fGAHT (Morssinkhof *et al.*, 2023). This is consistent with our findings after reinitiation, but not following discontinuation. The latter may be explained by three participants showing a steep decrease, likely influencing the group mean (see Supplementary Fig. S2C). Other sleep quality parameters and estradiol deprivation-related symptoms were consistent with findings in cisgender women during menopause (Sun *et al.*, 2014; Davis *et al.*, 2015; Zeng *et al.*, 2025). Sleep quality in postmenopausal women can be affected by frequent awakenings, often related to vasomotor symptoms such as hot flushes and night sweats (Zervas *et al.*, 2009). Our results similarly showed increased sleep disturbances, such as waking due to feeling too warm, after discontinuation and fewer after reinitiation.

Vasomotor symptoms and sleep problems can increase the risk of anxiety and depressive symptoms. Menopause in cisgender women is generally associated with poorer mental health, and lower QoL particularly among those experiencing estradiol deprivation-related symptoms compared to those without symptoms (Whiteley *et al.*, 2013; Davis and Baber, 2022). This contrasts with our findings, which showed no group-level differences on mental health or QoL. We also did not observe changes in body image, which typically improves after GAHT initiation and might be expected to decline with discontinuation (Meneguzzo *et al.*, 2024). Although these findings are encouraging, it is possible that more substantial changes in these outcomes may only emerge after a longer discontinuation period, highlighting the need for further research. Likewise, the slight decrease in happiness observed warrants further long-term investigation.

Interestingly, both the size of the changes and the number of affected outcomes were generally greater after reinitiation than after discontinuation. We anticipated that effects would balance out, with unfavorable changes after discontinuation generally mirrored by favorable changes after reinitiation. Insights from pain relief research may help explain these findings: while pain induces negative affect, alleviating pain produces a strong relief response by both a reduction in negative affect and an increase in positive affect (Franklin *et al.*, 2013). Although pain responses are typically more immediate, the underlying mechanism may be comparable. Thus, the greater effects after reinitiation may be attributed to a relief response resulting from the alleviation of symptoms experienced during discontinuation.

### Strengths and limitations

To our knowledge, this is the first clinical trial to address a range of psychological and physical outcomes following fGAHT discontinuation in older transgender women. The study was developed with input from older transgender adults, addressing questions important to both patients and clinicians, and providing a foundation for future research in this understudied area.

However, several limitations should be noted. The study was not blinded or placebo-controlled and relied on within-subject comparisons, which may have introduced bias due to participants’ expectations. While a blinded randomized controlled trial could better isolate the effects of estradiol deprivation, our design reflects the complex reality of fGAHT discontinuation for older transgender women. Furthermore, recruitment was challenging, likely due to the anticipated burden of the intervention, which may have resulted in a sample with more positive attitudes toward discontinuation and potentially underestimated negative experiences. Additionally, the study included only transgender women currently in treatment at the CEGD, excluding those who had already permanently discontinued fGAHT. Thus, our findings reflect the experiences of a clinical sample and may not generalize to the broader population of older transgender women. Last, the majority of participants were white, limiting insights into ethnic differences in estradiol deprivation-related experiences.

### Clinical implications

Transgender women, like cisgender women, experience symptoms in response to estradiol deprivation, which can negatively affect various aspects of life, including work, relationships, and QoL (Whiteley *et al.*, 2013; Davis and Baber, 2022). Estradiol deprivation-related symptoms are the primary indication for menopause hormone therapy in cisgender women, which is effective, particularly for vasomotor symptoms (Baber *et al.*, 2016; NICE, 2024). Therefore, transgender women who wish to continue fGAHT should be able to do so, with individualized risk assessment for conditions such as cardiovascular disease. At the same time, our study did not identify psychological or body image barriers to discontinuation, although individual variation must be considered. Thus, transgender women should have the option to discontinue fGAHT if desired.

However, it is important to note that this study did not address all potential medical risks associated with fGAHT discontinuation. All participants had undergone gonadectomy and therefore become hypogonadal following discontinuation. This increases the risk of osteoporosis and may also impact other health domains, such as cognitive functioning and cardiovascular disease, which are associated with estradiol deprivation in postmenopausal cisgender women (Davis *et al.*, 2015; Davis and Baber, 2022; Hogervorst *et al.*, 2022). The benefits and risks of fGAHT for these outcomes in older transgender women remain unclear. In contrast, transgender women with intact gonads who discontinue fGAHT may be re-exposed to endogenous testosterone production, likely mitigating some of these risks, particularly osteoporosis, but also potentially leading to masculinization. However, this depends on whether antiandrogens are used and continued, given that maintaining antiandrogens while discontinuing estradiol therapy would result in a similar hypogonadal state as experienced by the participants of this study. Therefore, individualized risk-benefit assessments for fGAHT discontinuation should be incorporated into clinical practice through a shared decision-making process. This process involves recognition by both clinician and patient that a decision needs to be made, consideration of patient preferences alongside clinician guidance, and a mutual understanding of the risks and benefits of the available options (Legare and Witteman, 2013). Achieving the latter remains challenging due to many unanswered questions in this population, underscoring the need for further research to be able to optimize decision-making in clinical practice.

### Future research

Due to feasibility constraints, the follow-up periods in this study were limited to 3 months. Long-term studies are needed to assess changes in mental health, QoL, and body image. Given the low participation rate and feedback from the patient advisory board, a long-term clinical trial with a large sample may not be feasible. Instead, observational studies including patients who choose to discontinue fGAHT independently could provide valuable insights into long-term well-being. Qualitative interviews with current study participants could offer a deeper understanding of the variation and appraisal of experiences. Additionally, we recommend that future research include testosterone dose reduction trials in transgender men, with study designs informed by focus groups and ongoing input from older transgender adults throughout the research process.

## Conclusions

The consequences and necessity of long-term continuation of fGAHT in older transgender women are not well understood. This study provides new insights, showing that short-term discontinuation resulted in estradiol deprivation-related symptoms, but did not indicate psychological or body image barriers to discontinuation for those who wish to stop. The variation in experiences and concerns about permanent discontinuation highlight the need for a personalized approach that focusses on patient preferences while considering somatic risk factors. Further research on the long-term risks and benefits of fGAHT in older age is essential to support optimal shared decision-making.

## Supplementary Material

deag087_Supplementary_Figure_S1

deag087_Supplementary_Figure_S2

deag087_Supplementary_Table_S1

## Data Availability

The data underlying this article cannot be shared publicly because participants did not provide written consent for their data to be shared.
