# Supplementary material for: Psychological and physical effects of short-term discontinuation of feminizing gender-affirming hormone therapy among older transgender women: a within-subject clinical trial
Source: Hum Reprod. 2026 May 30;41(8):1387–96. doi: 10.1093/humrep/deag087 (PMC13429873; doi:10.1093/humrep/deag087)
Supplement: deag087_Supplementary_Figure_S1 [file deag087_supplementary_figure_s1.pdf]

Human Reproduction, 2026, 1–1
https://doi.org/10.1093/humrep/deag087
Supplementary Data

human reproduction

OXFORD

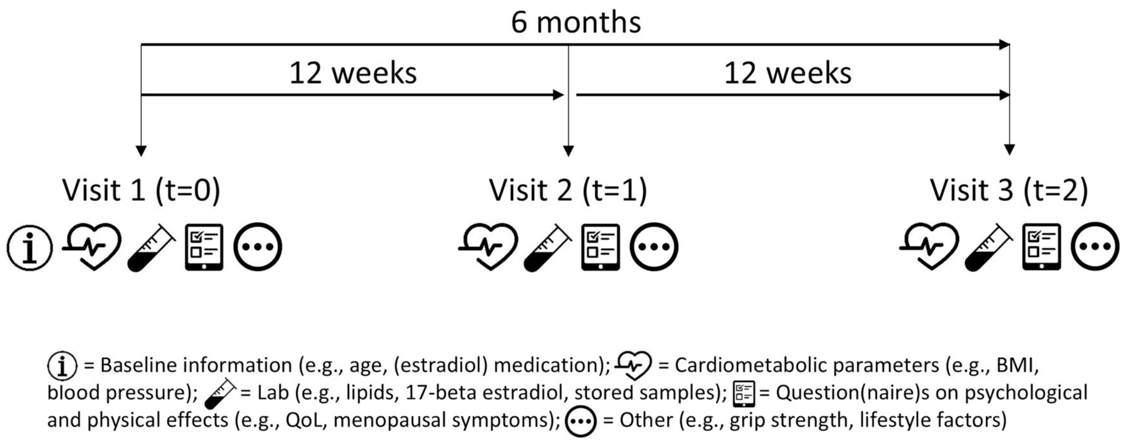

Supplementary Figure S1. Study design—overview of measurements per visit. t = 0 to t = 1: discontinuation and t = 1 to t = 2: reinitiation of feminizing gender-affirming hormone therapy. BMI: body mass index; QoL: quality of life.
