# Supplementary material for: Psychological and physical effects of short-term discontinuation of feminizing gender-affirming hormone therapy among older transgender women: a within-subject clinical trial
Source: Hum Reprod. 2026 May 30;41(8):1387–96. doi: 10.1093/humrep/deag087 (PMC13429873; doi:10.1093/humrep/deag087)
Supplement: deag087_Supplementary_Figure_S2 [file deag087_supplementary_figure_s2.pdf]

Human Reproduction, 2026, 1–1
https://doi.org/10.1093/humrep/deag087
Supplementary Data

human reproduction
OXFORD

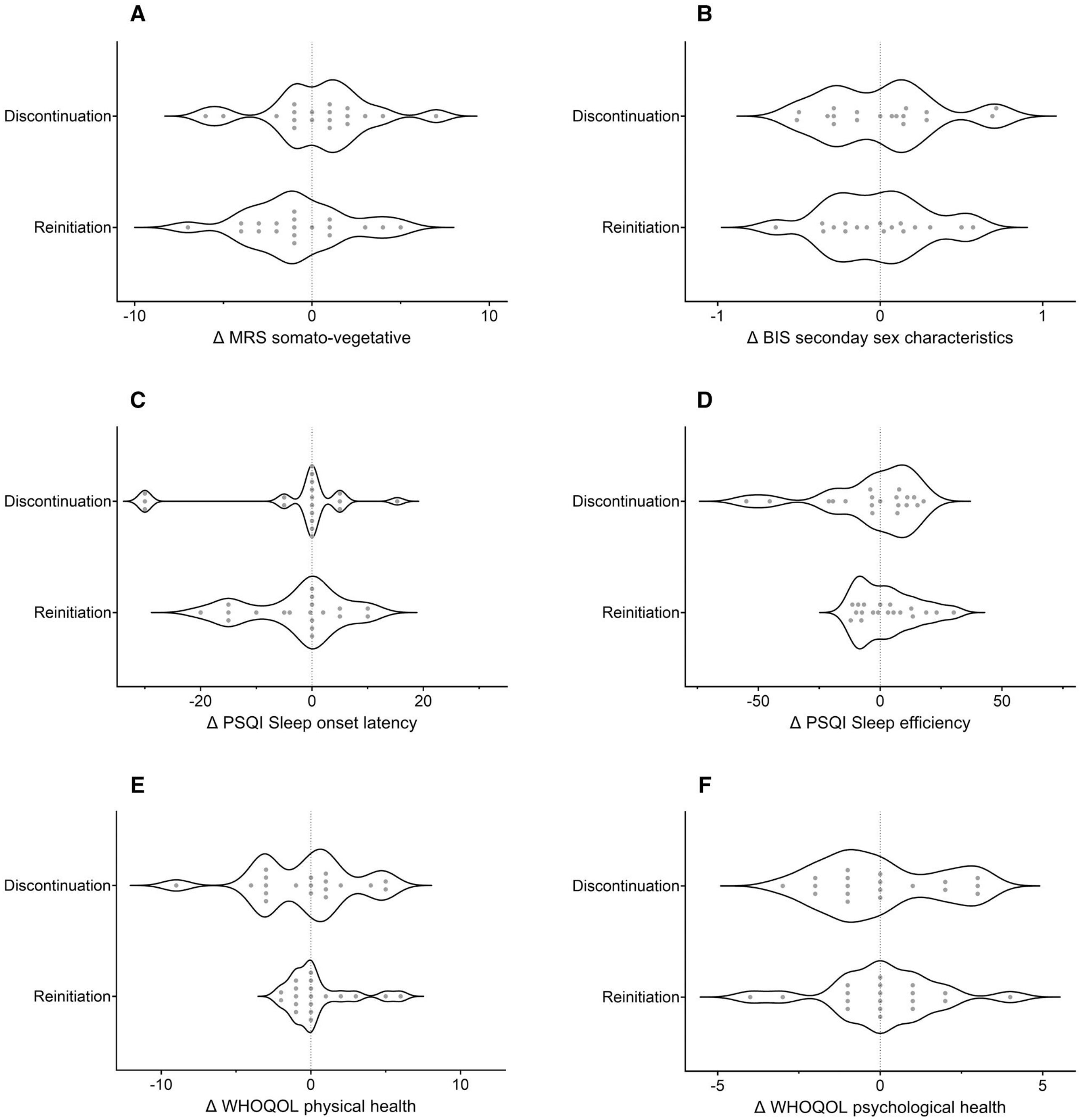

Supplementary Figure S2. (A-F). Violin plots showing the variation in change over time on outcome subscores. Each point represents the change score of one participant, with ‘discontinuation’ showing the difference between $t = 0$ and $t = 1$, and ‘reinitiation’ between $t = 1$ and $t = 2$. The dotted lines at zero indicate no difference. Δ: score differences; MRS: Menopause Rating Scale; BIS: Body Image Scale; PSQI: Pittsburgh Sleep Quality Index; WHOQoL: World Health Organization Quality of Life.
