# Supplementary material for: Psychological and physical effects of short-term discontinuation of feminizing gender-affirming hormone therapy among older transgender women: a within-subject clinical trial
Source: Hum Reprod. 2026 May 30;41(8):1387–96. doi: 10.1093/humrep/deag087 (PMC13429873; doi:10.1093/humrep/deag087)
Supplement: deag087_Supplementary_Table_S1 [file deag087_supplementary_table_s1.pdf]

Human Reproduction, 2026, 1–1
https://doi.org/10.1093/humrep/deag087
Supplementary Data

human reproduction

OXFORD

Supplementary Table S1. Sensitivity analyses excluding outliers, showing estimated means, mean differences, and percentage change between measurements following short-term discontinuation ($t = 0$ to $t = 1$) and subsequent reinitiation ($t = 1$ to $t = 2$) of feminizing gender-affirming hormone therapy.

	Original analyses		Excluding outliers (>1.5*IQR)		Original analyses		Excluding outliers (>1.5*IQR)	
**Outcome variables**	**Estimated mean difference $t = 0$ to $t = 1$ (95% CI)**	**% change**	**Estimated mean difference $t = 0$ to $t = 1$ (95% CI)**	**% change**	**Estimated mean difference $t = 1$ to $t = 2$ (95% CI)**	**% change**	**Estimated mean difference $t = 1$ to $t = 2$ (95% CI)**	**% change**
Estradiol deprivation-related symptoms—Total	0.2 (−1.6 to 2.0)	2.6	–	–	**−3.0 (−4.8 to −1.2)**	**−36.3**	**−3.3 (−5.2 to −1.4)**	**−40.2**
Somato-vegetative symptoms	0.4 (−0.7 to 1.5)	**11.5**	–	–	−0.8 (−1.9 to 0.3)	**−22.1**	–	–
Sleep quality—Total	0.4 (−0.4 to 1.3)	6.8	0.6 (−0.2 to 1.5)	**11.0**	−0.7 (−1.5 to 0.2)	**−10.3**	−0.8 (−1.7 to 0.0)	**−13.5**
Sleep onset latency	−3.7 (−9.0 to 1.6)	**−16.6**	–	–	−2.8 (−8.1 to 2.5)	**−14.9**	−4.0 (−9.6 to 1.6)	**−21.9**
Sleep efficiency	−3.7 (−11.0 to 3.5)	−4.8	−5.6 (−12.3 to 1.1)	−7.0	2.7 (−4.6 to 9.9)	3.6	4.4 (−2.4 to 11.1)	5.9
Sleep disturbance	1.6* (0.2 to 10.3)	**10.7**	–	–	**0.1* (0.0 to 1.0)**	**−46.6**	–	–
Depressive symptoms	0.1 (−1.3 to 1.4)	0.4	–	–	−1.1 (−2.4 to 0.3)	−7.7	–	–
Anxiety symptoms	0.2 (−1.6 to 2.0)	1.2	–	–	−1.0 (−2.8 to 0.8)	−7.3	–	–
Happiness	**−0.5 (−0.9 to −0.1)**	−6.4	**−0.6 (−1.0 to −0.1)**	−7.5	0.2 (−0.2 to 0.6)	3.1	–	–
QoL—Physical health	−0.5 (−1.7 to 0.7)	−1.8	–	–	0.4 (−0.8 to 1.6)	1.7	–	–
QoL—Psychological health	0.0 (−0.8 to 0.8)	0.0	–	–	0.1 (−0.8 to 0.9)	0.2	–	–
Body image—Total	0.0 (−0.1 to 0.2)	0.7	−0.1 (−0.2 to 0.1)	−1.9	−0.1 (−0.2 to 0.1)	−2.0	0.0 (−0.1 to 0.1)	−0.9
Body image—Secondary sex characteristics	0.0 (−0.1 to 0.2)	0.3	0.0 (−0.2 to 0.1)	−0.2	0.0 (−0.2 to 0.1)	−1.5	–	–

Bold = significance based on 95% CI or >10% change in estimated means between measurements.
Outcome variables were measured using the following questionnaires, listed in order from top to bottom: Menopause Rating Scale, Pittsburgh Sleep Quality Index, PROMIS depression Short Form v1.0 8b, PROMIS anxiety Short Form v1.0 8a, Cantril ladder, World Health Organization QoL-BREF, Body Image Scale.
* Dichotomous outcome, therefore, odds ratios are show.
IQR: interquartile range; QoL: quality of life.
